# *Drosophila* Models of Tauopathies: What Have We Learned?

**DOI:** 10.1155/2012/970980

**Published:** 2012-06-04

**Authors:** Marc Gistelinck, Jean-Charles Lambert, Patrick Callaerts, Bart Dermaut, Pierre Dourlen

**Affiliations:** ^1^Laboratory of Behavioral and Developmental Genetics, Center for Human Genetics, University of Leuven, 3000 Leuven, Belgium; ^2^VIB Center for the Biology of Disease, 3000 Leuven, Belgium; ^3^INSERM U744, Institut Pasteur de Lille, Université Lille Nord de France, 1 Rue du Professeur Calmette, 59019 Lille Cedex, France

## Abstract

Aggregates of the microtubule-associated protein Tau are neuropathological hallmark lesions in Alzheimer's disease (AD) and related primary tauopathies. In addition, Tau is genetically implicated in a number of human neurodegenerative disorders including frontotemporal dementia (FTD) and Parkinson's disease (PD). The exact mechanism by which Tau exerts its neurotoxicity is incompletely understood. Here, we give an overview of how studies using the genetic model organism *Drosophila* over the past decade have contributed to the molecular understanding of Tau neurotoxicity. We compare the different available readouts for Tau neurotoxicity in flies and review the molecular pathways in which Tau has been implicated. Finally, we emphasize that the integration of genome-wide approaches in human or mice with high-throughput genetic validation in *Drosophila* is a fruitful approach.

## 1. Introduction

For more than a century, the fruit fly *Drosophila *has been used to unravel major biological questions. The fruit fly has played crucial roles in deciphering various developmental signaling cascades such as the Notch, Wingless, and Hedgehog pathways. In addition, studies using *Drosophila* have contributed to a wide range of topics in neurobiology including neurodevelopment, behavior, circadian rhythms, learning and memory, synaptic transmission, and neurodegeneration [1, 2]. Since most basic molecular and cell biological mechanisms are conserved between humans and *Drosophila *and since *∼*70% of all human disease genes have an evolutionary conserved fly homolog, studies in flies have also provided valuable insights into the biology of human disease [3]. During the last decade, *Drosophila* has gained attention as a model system for common human neurodegenerative brain disorders [4]. In general, these models are based on the misexpression of human proteins such as *α*-synuclein [5], Tau [6, 7], and TDP-43 [8] that are present in the neuropathological hallmark lesions of patients with Parkinson's disease (PD), Alzheimer's disease (AD), frontotemporal dementia (FTD), and amyotrophic lateral sclerosis. Interestingly, expression of these proteins in flies results in neurotoxicity and the underlying molecular mechanisms appear to be largely protein- or disease-specific suggesting that this approach is useful.

Here, we review the contribution of *Drosophila *to the molecular understanding of Tau neurotoxicity, a central player in the AD-FTD spectrum of disorders [9]. We give a brief overview of the most commonly used genetic tools in *Drosophila* and summarize the different available Tau models and readouts for Tau neurotoxicity. Together, these studies paint a multifaceted picture of Tau being involved in a wide range of biological processes and highlight the complex role of Tau phosphorylation in mediating its toxicity.

## 2. Modeling Tauopathy in *Drosophila*

### 2.1. The *Drosophila* Genetic Toolkit in a Nutshell

Next to the fact that the fundamental molecular and cell biological aspects of neuronal biology are conserved between human and *Drosophila*, the main advantage of the fly is its powerful, flexible, and extensive genetic toolkit. It essentially allows the expression, downregulation, or mutation of any gene, in a tissue- and time-specific manner [10].

A major tool is the binary GAL4-UAS system, which allows the expression of a genetic responder construct downstream of an Upstream Activator Sequence (UAS) driven by tissue-specific expression of the yeast GAL4 transcription factor [11]. The system can be used to either silence a gene using an RNAi-construct or to induce mis- and/or overexpression using a cDNA construct. Many driver lines have been generated, in which promoters of genes have been inserted upstream of the GAL4 sequence. The diversity of gene promoters makes it possible to target nearly all tissue or cell types. However, they usually do not allow time specificity. The most commonly used GAL4 drivers induce expression of the target protein from early developmental stages. Restricting expression to adulthood or to a defined time is relevant when modeling late-onset degeneration. For this reason, modifications of the GAL4-UAS system were developed in *Drosophila* and allow a tight regulation of transgene expression. For example, the TARGET system uses a temperature-sensitive mutant of the yeast GAL4 repressor GAL80 [12]. GAL80^ts^ is active at low temperatures and suppresses GAL4 activity. To activate GAL4-induced gene expression in adulthood, adult flies are moved to 30°C, a temperature at which GAL80^ts^ becomes inactivated and no longer inhibits GAL4 activity. In addition expression is reversible and shut off when shifting the flies back to lower temperature. Another system of conditional gene expression in *Drosophila* is called the Geneswitch system [13]. It consists of the pharmacological activation of a RU486-sensitive GAL4-derived transcription activator. The yeast GAL4 DNA binding domain has been fused with a mutated human progesterone receptor-ligand binding domain and with the transcriptional activation domain of the human p65, a member of the NFkB family [14]. The chimeric fusion protein is activated by RU486, binds to UAS sequences, and activates the transcription of downstream sequences. For RU486 induction, RU486 is added to *Drosophila* food [13].

Among the other genetic tools of *Drosophila* are transposons, which are mobile genetic elements, in which the transposase has been replaced by other sequences, such as UAS sequences to generate enhancer traps and GFP sequence that can be spliced to generate protein traps for example [10]. The main advantage is that these elements can be easily mobilized and insert randomly in the genome. If the transposon disrupts the gene sequence in which it is inserted, it can generate *loss-of-function* alleles of the gene. The imprecise excision of the transposon can also be used to generate genomic null mutations [10]. Different transposons such as P-element, PiggyBac, or Minos elements with complementary bias in their insertion site are now used to cover the whole *Drosophila* genome [15]. Null alleles can also be generated by chemical mutagenesis or X-ray radiation. Other powerful techniques are based on mitotic recombination, which can be used in a controlled manner to generate homozygous-mutant tissue in a heterozygous background. This allows determining the function of developmentally lethal genes in adult tissues [16]. All these tools give to researchers using *Drosophila* the possibility to perform in-depth reverse genetic studies as well as large-scale forward genetic screens, enabling the identification of novel biological pathways in an unbiased manner [17].

### 2.2. Tau Genetic Reagents

At least 37 constructs have been used to generate transgenic *Drosophila* Tau strains (Table 1). Tau cDNAs are most frequently inserted downstream of a UAS promoter although some Tau cDNAs are inserted downstream of the eye-specific *gl* promoter enabling simultaneous and independent expression of other UAS-constructs [7]. Tau transgenes were first used to improve neuronal labeling in morphological studies [18–20] until Williams and coworkers showed that these constructs induce neurodegeneration characterized by axonal loss and swellings [21]. Many *Drosophila* models were then generated using human Tau (hTau) (Table 1). Some are based on 0N3R, 0N4R, and 2N4R wild-type hTau isoforms [6, 7, 21], whereas others express mutated forms hTau, that cause autosomal dominant Tau-positive FTD, such as hTau^R406W^, hTau^V337M^, and hTau^P301L^ [6, 22]. To explore specific mechanisms of hTau toxicity or dysfunction, transgenes with targeted mutations and truncations were also generated, including constructs which abolish or mimic hTau phosphorylation or proteolytic cleavage [23–31]. Together, these models explore the great diversity of tauopathies.

While most *Drosophila* studies on Tau neurotoxicity are based on overexpression of hTau, it is important to mention that *Drosophila* has a single *tau* gene/protein (dTau) [36]. Compared to the 6 human isoforms, which harbor either 3 or 4 C-terminal microtubule-binding domains (MTBD) and 0 to 2 N-terminal insertions, the dTau protein contains 5 MTBD with 46% identity and 66% similarity to the corresponding hTau region but no N-terminal insertions [36]. Homozygous dTau null *Drosophila* mutants are viable and fertile and display no obvious morphological or behavioral defects [33], although a microtubule-based defect in polarity has been shown in dTau null oocytes [37]. The absence of major defects in dTau null mutants may be due to redundancy with other microtubule-associated proteins such as Futsch, the MAP1B *Drosophila* ortholog. In agreement, the degenerative phenotype of hypomorphic *futsch* alleles was partially suppressed by dTau overexpression in the central nervous system [38]. Some constructs also express dTau, which have been used to compare the function and toxicity of endogenous dTau with hTau [32–34, 39]. These studies revealed similar degrees of neuronal dysfunction for dTau and hTau (see below) although genetic and physical interaction partners showed important differences between the two Tau proteins [39, 40].

### 2.3. Readouts of Tau Neurotoxicity and Dysfunction in *Drosophila*

The choice of a readout for Tau neurotoxicity or dysfunction results from a trade-off between the ease and speed of scoring a phenotype and its biological or pathogenic relevance. The *Drosophila* exoskeleton provides a wealth of external features, such as bristles and compound eyes, which can be affected by genetic manipulations, and for which the resulting phenotypes can be scored simply and quickly in young flies by looking through a stereomicroscope. These readouts of neurotoxicity or dysfunction, especially the eye external morphology, have been successfully used in screens for modifiers of Tau pathology and other neurodegenerative diseases [41–43]. Here we give an overview of the different available readouts of Tau neurotoxicity and dysfunction in *Drosophila* (Table 2).

#### 2.3.1. The Eye

Roughening of the eye is the most commonly used external phenotype to evaluate toxicity of neurodegenerative proteins, including Tau, in *Drosophila*. The fly eye consists of around 800 highly regularly implanted ommatidia, each containing 8 photoreceptor neurons. The eye is an excellent tissue to study the effect of organismal lethal genes as it is dispensable for viability. Due to its repetitive crystal-like pattern, it is ideal to identify mild external morphological defects upon expression of human neurotoxic proteins. It thus constitutes a genetically sensitized system that allows the identification of genetic modifiers by assessing roughening of the eye as a quantitative readout of neurotoxicity. The eye surface of the *Drosophila* eye is generated during the final stages of development and thus, this phenotype has a developmental component. However, it is associated with vacuolization in the underlying optic brain structures [7], a typical sign of degeneration in the fly nervous system. Interestingly, a pure degenerative phenotype can be established in the *Drosophila* eye by assessing the viability of photoreceptor neurons over time in living adult flies. This is possible thanks to the cornea neutralization technique, which consists of visualizing photoreceptor neurons directly through the cornea of a living anesthetized fly, immersed in a medium with a refractive index comparable to the fly cornea, such as water [44]. Photoreceptor neurons are detected based on Rhodopsin autofluorescence or on the expression of fluorescent protein such as GFP. Because it is an *in vivo* method, the same fly can be analyzed at several time points during adult lifespan and exhibit progressive degeneration. The usefulness of related fluorescence-based techniques in neuronal degeneration was further illustrated by Gambis, and colleagues [45]. In a clonal screen using a derived method, called Tomato/GFP-FLP/FRT method, several mutants were identified that induced progressive photoreceptor loss. These methods have not yet been tested in the context of Tau neurotoxicity or other proteinopathies. Another potentially useful method that has not yet been used in *Drosophila* models of Tauopathies is the electrophysiological analysis using electroretinogram recordings (ERG). ERG analysis consists of recording the electrophysiological activity of the retina upon exposure to light. This activity is sensitive to PR degeneration. ERG measurements can be used to show a progressive loss of neuronal functioning [46].

#### 2.3.2. The Notal Bristles

Neurotoxicity assays based on external fly features, such as eye roughness, are important as they are easily scorable and thus suited for high-throughput genetic screening. Interestingly, a novel bristle loss phenotypic assay for Tau neurotoxicity was described recently [47]. The *Drosophila* notum (part of the thorax) harbors around 200 bristles, which are sensory organs, connected at the base with the dendrite of a sensory neuron. Overexpression of different variants of hTau in the notum using the notum-specific Eq-GAL4 driver leads to bristle loss. hTau toxicity can be quantified by simply counting the bristles. This was done for wildtype hTau, phosphomutant hTau, and phosphomimetic hTau in addition to FTD-associated mutant hTau. In general, the sensitivity of the eye and the bristles to different variants was comparable. The notal bristle assay thus constitutes an interesting complementary and quantitative model to molecularly dissect Tau neurotoxicity.

#### 2.3.3. Lifespan and Lethality

The ultimate consequence of (neuro)toxicity is the death of the whole organism, which can also be scored relatively easily by counting the number of surviving flies over time. Depending on the rearing conditions, wild-type flies live around 60 to 80 days and lifespan can be easily used as a quantifiable readout of Tau toxicity [6, 31, 47–50]. On the other hand, lethality can occur during development before eclosion of adult flies. Using the pan-neuronal Appl-GAL4 and Elav-GAL4 drivers, Tau-expressing flies exhibited pupal lethality, which can also be quantified and serve as a readout of Tau toxicity [49, 51, 52].

#### 2.3.4. Brain Degeneration

Neuronal degeneration in *Drosophila* can be demonstrated by the presence of vacuoles in brain tissue using histological and immunohistochemical methods. Interestingly, vacuoles are found in the brains of hTau-expressing flies [6, 7, 32] and, although labor-intensive, the number of vacuoles can be used as a quantitative readout of hTau neurotoxicity [29, 53, 54]. Neuronal cell death can be further detected using specific stainings. The TUNEL technique is frequently used to detect apoptosis in brains of hTau-expressing flies [25, 29, 50, 54]. Alternatively, immunostaining of activated cleaved caspase-3 can be performed to demonstrate apoptotic cell death [50, 53].

#### 2.3.5. Axonal Transport Assays

The main function of Tau is to bind to microtubules. Hence, it has been hypothesized that Tau toxicity or dysfunction could result from a defect in axonal transport. *Drosophila* is well suited to study axonal transport because fluorescent (GFP) fusion proteins tagging transport vesicles can be expressed in larval motor neurons, which are accessible for imaging in living intact animals [55]. In addition, larval locomotor phenotypes have been described for mutants that affect axonal transport, such as kinesin or dynein mutants [56, 57]. Immunostainings against Synaptotagmin were first performed to assess axonal transport in Appl-GAL4 larvae expressing hTau [51, 52]. It enables the visualization of synaptic vesicles along axons. Mudher and colleagues then used a GFP/neuropeptideY fusion protein to image vesicle axonal transport through the body wall of living larva expressing hTau in motor neurons (D42-GAL4 driver) [40, 48, 55]. Talmat-Amar and colleagues further enriched the analysis with a Synaptotagmin-GFP construct expressed in larval motor neurons (OK6-GAL4 driver) and with kymographs [49]. Kymographs consist of visualizing the movements of all vesicles within a nerve segment over time. They allow the measurement of the kinetic parameters of vesicular movement such as instant velocity or pausing time.

Axonal transport disruption can also be detected at the level of the whole organism. Two readouts, locomotor deficits and the “juvenile” phenotype, have been used depending on the neurons in which hTau is expressed. First, Mudher and colleagues analyzed successfully the motor function of larvae expressing hTau in motor neurons (D42-GAL4) using contraction, crawling, line-crossing, and righting assays [55]. These assays enabled them to compare the effects of 3R hTau and dTau on locomotor functions or the interaction between hTau and A*β*42 [40, 48]. Locomotor functions can also be assessed in adult flies using a climbing or negative geotaxis assay. Flies display a strong negative geotactic response. When tapped to the bottom of a vial they rapidly climb to the top of the vial, and most flies remain there. Locomotor dysfunction impairs climbing ability. Using this readout, locomotor dysfunction was quantified in Tau-expressing flies (D42-GAL4 and elav-GAL4) [53, 55]. Second, a strong defect in axonal transport can also result in altered release of neuropeptides or neurohormones at neurohemal release sites. When bursicon neurons are affected, loss of the bursicon neurohormone prevents wing expansion and cuticle tanning just after fly eclosion [58]. This immature unexpanded wing phenotype is easily visible and quantifiable in young flies. Several drivers, OK6-GAL4, Burs12-GAL4, Appl-GAL4 or elav-GAL4, are expressed in bursicon neurons [51, 59, 60], and it has been shown that the inhibition of axonal transport *per se* in bursicon neurons affects wing expansion [49]. This phenotype has been used as a readout to compare the toxicity of phosphorylation mutants of hTau and to show a genetic interaction between hTau and *Appl, *the fly APP homolog [49, 51].

#### 2.3.6. Learning and Memory Assays and Mushroom Body Ablation Phenotypes

Tauopathies affect the cognitive functions of patients. In *Drosophila*, olfactory memory can be used as a readout for assessing impaired cognitive functions in hTau*-*expressing flies. Olfactory learning and memory relies on neurons located in a distinct region of the fly brain called mushroom bodies [61]. Tau expression can be targeted to these neurons using the pan-neuronal driver elav-GAL4 or the late pupal, adult mushroom body-specific drivers C492-GAL4, C772-GAL4 [24, 32]. By testing response to attractive and repulsive odors, olfactory learning and memory has been standardized and can be measured in transgenic flies. An aversive phototaxis suppression assay has also been used to measure learning and memory function in Tau-expressing flies [32, 53]. Strikingly, pan-neuronal overexpression of hTau leads to selective and nearly complete ablation of the mushroom bodies [24, 32]. These phenotypes can also be used to identify genetic interactors of Tau dysfunction/toxicity.

## 3. Pathogenic Mechanisms of Tauopathies

### 3.1. Tau and Phosphorylation

Studies in *Drosophila* have revealed a highly complex role of Tau phosphorylation in mediating neuronal function or toxicity. The rough eye phenotype was the starting point for the Jackson lab, to investigate the role of Wingless signaling and *Shaggy* (*Sgg*, the fly GSK3*β* homolog) on hTau toxicity [7]. Overexpression of *Sgg* significantly enhanced hTau toxicity, even to the point that neurofibrillary tangle-like structures could be detected, while a loss-of-function allele of *Sgg* had a beneficial effect on toxicity. In order to investigate if hTau toxicity is mediated by Wingless signaling, genetic interaction between Tau and the downstream Sgg target *armadillo* (*arm*), which is inhibited by Sgg, was investigated. Unexpectedly, loss of *arm* rescued whereas misexpression enhanced the hTau rough eye phenotype independently of hTau phosphorylation. These results suggested that the *Sgg* interaction with hTau does not go through the canonical Wnt pathway and that Sgg directly or indirectly leads to phosphorylation of hTau (see also Section 3.5.1). A follow-up study further suggested an indirect effect since the mutant 2N4R hTau^S11A^ that cannot be phosphorylated by Sgg is still toxic [23].

The role of phosphorylation in Tau pathology was investigated by several labs [23, 25–27, 49, 62]. The Lu lab showed that, overexpression of fly PAR-1 kinase (MARK) in the eye induces a moderate eye phenotype, which was partially suppressed in a heterozygous deletion background of dTau. A strong synergistic enhancing effect was observed in a background expressing hTau^R406W^ [25]. Reduction of PAR-1 function or mutation of PAR-1 phosphorylation sites (S2A) was shown to reduce 0N4R hTau^R406W^ toxicity. Phosphorylation was also suggested to occur in a structurally ordered pattern. First S262 and S356 are phosphorylated which facilitates targets of Sgg to be phosphorylated. The Jackson lab independently showed that wild-type 2N4R hTau, in which serines S262 and S356 are substituted to alanines (S2A), displayed lower toxicity in the eye due to the inability of PAR-1 to phosphorylate the S2A tau at the two mutated serines [48]. However, the Lu and Jackson labs disagree about the priming effect of PAR-1 before Sgg phosphorylation. The Lu lab showed that the 0N4R hTau^R406W/S2A^ construct is not phosphorylated at some Sgg sites and is refractory to toxic enhancement by Sgg overexpression [25], while the Jackson lab found that 2N4R hTau^S2A^, although less toxic, was still phosphorylated at Sgg sites when Sgg is overexpressed [23]. The different conclusions by the two labs are unclear but may be due to the different isoforms used (0N4R hTau^R406W/S2A^ versus 2N4RhTau^S2A^). Altogether, these results demonstrate, in contrast to Sgg, a direct role for PAR-1 in mediating Tau toxicity.

In order to identify phosphorylation sites involved in toxicity, the Feany lab generated a number of 0N4R hTau constructs each having one or two Ser-Pro and/or Thr-Pro mutated to phosphoresistant alanines (Table 1) [27]. Although no significant reduction in toxicity was observed for any of these phospho-deficient constructs, a 0N4R hTau construct in which 14 Ser-Pro and Thr-Pro target sites are mutated to alanine (hTau^AP^) displayed reduced neurotoxicity [26]. Accordingly, a phosphomimetic construct with the same 14 epitopes mutated to glutamate (hTau^E14^) increased Tau toxicity, suggesting that toxicity relies on cooperation of different phosphorylation sites. However, using a 2N4R construct in which 11 Ser-Pro and Thr-Pro target sites are mutated to alanine (hTau^S11A^) of which 9 overlapped with hTau^AP^, the Jackson lab did not observe decreased toxicity in the eye [23]. The reasons for these discrepancies are not clear but might be related to the different hTau isoforms used, the number of mutated sites, or the differences in the mutated sites. The toxicity of the phosphomimetic Tau^E14^ construct has been further reported in mushroom body neuroblasts, whereas the phospho-deficient Tau^AP^, 0N4R hTau^R406W/S2A^, and 2N4R hTau^S2A^ were not toxic [24]. In mushroom body neuroblasts, Ser238 and Thr245 were also shown to be essential for 2N4R hTau toxicity [24].

Tau phosphorylation also affects neuronal function in the absence of neuronal loss/toxicity. The tau protein is known best, functionally, as a microtubule stabilizing protein. The fraction of Tau that is bound to microtubules is inversely correlated with its phosphorylation state. In the context of microtubule stabilization, hyperphosphorylation leads to *loss-of-function *effects while hypophosphorylation can lead to increased microtubule stabilization. How expression of hyperphosphorylated hTau affects microtubular integrity in the fly was elegantly investigated by the Mudher group [55, 63]. They found that, despite the presence of endogeneous dTau, overexpression of 0N3R hTau led to axonal microtubule breakdown. When expressed in flies, 0N3R hTau becomes hyperphosphorylated. Upon treatment with Li^+^, a GSK3*β* inhibitor, phosphorylation was reduced and microtubule binding of hTau was increased. Interestingly, the same was shown for dTau, suggesting that hyperphosphorylated hTau can sequester endogeneous dTau. Furthermore, it was shown that the physical interaction between dTau and hTau occurred in a phosphorylation-dependent manner. In addition, the Mudher lab found that overexpression of hTau in motorneurons leads to defects in axonal transport without neurodegeneration. They investigated the role of hTau phosphorylation by Sgg on axonal transport. Pharmacologic inhibition of Sgg, with two different inhibitors, could reverse Tau-induced defects in axonal transport. Overexpression of hTau resistant to Sgg phosphorylation (Tau^S11A^) was later shown to display a higher binding affinity for microtubules [23]. This suggests that pharmacologic inhibition of Sgg probably restores axonal transport by increasing the microtubule affinity of hTau. Later, Talmat-Amar and coworkers showed that at the level of axonal transport, hTau^AP^ was clearly more toxic than hTau^WT^ or hTau^E14^ and related to its capacity to bind more strongly to microtubules [49]. Taken together, these data suggest that microtubule affinity of hTau is most likely, at least in part, regulated by Sgg-dependent phosphorylation. Furthermore, it can be concluded that both hypo- and hyperstabilization of axonal microtubules has detrimental effects.

### 3.2. Tau and the Cytoskeleton

#### 3.2.1. Binding of Tau to Microtubules

By nature, Tau is a microtubule-binding protein and therefore has a strong involvement in the regulation of the cytoskeleton. In *Drosophila,* endogenous dTau and exogenous bTau and hTau colocalize with microtubules *in vivo* [33, 35, 36]. dTau and hTau interact with microtubules in microtubule cosedimentation assays *in vitro* [23, 33]. dTau binds microtubules more strongly than 0N4R hTau or hTau^R406W^ and hTau^V337M^ [34]. This weak binding of hTau to microtubules depends on its phosphorylation status. The microtubule-bound hTau in the pellet was found to be hypophosphorylated, whereas hTau in the supernatant was phosphorylated [34]. In addition, using phosphomimetic and phospho-deficient hTau forms to assess the role of phosphorylation, it was shown that the majority of phospho-deficient hTau^AP^ and hTau^S11A^ proteins were pelleted with microtubules, whereas the pseudophosphorylated Tau^E14^ proteins remained mostly in the supernatant fraction [23, 34, 49]. Thus hTau expressed in *Drosophila* is phosphorylated, which prevents a strong binding to microtubules.

#### 3.2.2. Functional Consequences of Deregulated Tau-Microtubule Interaction

One functional consequence of Tau deregulation is the alteration of the microtubule network. Overexpression of hTau has been associated with microtubule breakdown in peripheral nerves of L3 larvae [63]. In this model, the cytosolic phosphorylated hTau bound endogenous dTau and dissociated dTau from microtubules. This would be responsible for the disruption of the microtubule cytoskeleton as claimed by the Tau-microtubule hypothesis [64]. In loss-of-function experiments, both follicle and germline cells of dTau null ovaries did not display strong alterations in their microtubule network [33]. However, the polarity of dTau null oocytes was altered at stage 10, a phenotype similar to that of excessive PAR-1 overexpression [37]. In addition, dTau overexpression partially rescued the phenotype of PAR-1 overexpression, which suggests that dTau is involved in maintaining microtubule stability in the oocyte and that PAR-1 regulates oocyte polarity at least partly through dTau [37].

Another functional consequence of Tau deregulation is the alteration of axonal transport. Overexpression of bTau, 0N3R hTau, and dTau in third instar larvae results in large accumulations of synaptotagmin or GFP:neuropeptideY-tagged vesicles in motor neuron axons [40, 51, 55]. Coexpression of GSK3*β* and pharmacological inhibition of GSK3*β*, respectively, increased and decreased this phenotype, suggesting that Tau phosphorylation could enhance axonal transport disruption [55]. Although this study did not detect a vesicle motion defect, a later study also using 0N4R hTau showed an increase in the pausing rate of the vesicles within axons [49]. The pausing defect was drastically stronger using 0N4R hTau^AP^, a phospho-deficient form that strongly binds to microtubules and affected mainly anterograde transport. Expression of hTau^AP^ induced a juvenile wing inflation phenotype, similarly as downregulation of dynein and kinesin [49]. Expression of the pseudophosphorylated hTau^E14^ did not disrupt vesicle motion and even slightly increased instant velocity [49]. Interestingly, a reciprocal regulation of Tau phosphorylation by axonal transport has been described [52]. A nonlethal reduction in kinesin-1-dependent axonal transport was associated with increased activated cJun N-terminal Kinase (JNK), hTau phosphorylation, hTau accumulation, axonal vesicle accumulation, and hTau toxicity [52]. This suggested that axonal transport defects can activate axonal stress kinase pathways leading to hTau phosphorylation, stabilization, and an increase in hTau-mediated neurodegeneration.

 Taken together, these studies seem to converge on the notion that direct PAR-1-mediated Tau phosphorylation is directly involved in neurotoxicity, while the effects of GSK3*β* appear to be more indirect and related to neuronal functioning rather than toxicity.

#### 3.2.3. Tau and the Actin Cytoskeleton

*cheerio* (fly ortholog of filamin), *chd64* (fly ortholog of transgelin-3), *jaguar* (fly ortholog of myosinVI), *paxillin*, 4 regulators of the actin network, were identified as modifiers of the Tau^V337M^-mediated rough eye phenotype in a misexpression screen [43]. *cheerio* had been identified also in a previous similar screen [41]. Filamin-A and MyosinVI were further found to colocalize with fibrillary hTau protein in AD and FTD brains [65]. The Feany lab showed that actin might be a critical mediator of Tau-induced neurotoxicity [66]. They showed that hTau^R406W^ interacts directly with F-actin in the fly brain. hTau^R406W^ overexpression induced the accumulation of F-actin and the formation of actin-rich rods, which were similar to Hirano bodies found in AD. F-actin accumulation and the formation of actin-rich rods correlated with the degree of Tau-induced neuronal degeneration. Decreasing F-actin levels reduced neurotoxicity in the retina of hTau transgenic flies. This indicated that F-actin mediated hTau neurotoxicity [66]. In addition, whereas hTau^E14^-induced retinal toxicity was clearly modified by genetically modulating the actin cytoskeleton, the hTau^AP^-induced rough eye was not enhanced by coexpressing actin. This showed that actin changes occur downstream of hTau phosphorylation [66].

#### 3.2.4. Tau and the Larval Neuromuscular Junction (NMJ)

It has been shown that overexpression of dTau, 0N3R hTau, 0N4R hTau, and hTau^V337M^ in larval motor neurons causes morphological disruption of NMJs characterized by satellite boutons [40, 43, 67]. In 0N3R hTau-expressing motor neurons, this is associated with abnormal endo/exocytosis characterized by decreased evoked synaptic potentials following high frequency stimulation [67]. The authors suggested that this may be due to a reduction in axonal transport of mitochondria resulting in a reduction of functional mitochondria in the presynaptic terminal [67]. No axonal transport defects were observed in hTau^V337M^-expressing larval motor neurons but in this model, abnormally shaped NMJs were associated with loss of acetylated alpha-tubulin [43]. The authors suggested that disruption of the cytoskeleton network in presynaptic nerve terminals could constitute early events in the pathological process leading to synaptic dysfunction in hTau^V337M^ pathology.

### 3.3. Degradation of Tau

Dysfunction of protein degradation may favor accumulation of toxic Tau species. Several studies in *Drosophila* have analyzed Tau degradation. Endogenous dTau was first described as being not degraded by the proteasome pathway [68] although high-molecular-weight forms of hyperphosphorylated hTau were shown to be degraded by the proteasome [43]. *Hsp70/Hsp90-organizing protein homolog* (*Hop*), a scaffold protein for chaperones, has been identified as a suppressor of 2N4R hTau toxicity and has been proposed to facilitate clearance of hTau via the Ubiquitin-Proteasome System (UPS) [42]. Recently, nicotinamide mononucleotide (NAD) adenylyl transferase (NMNAT), a protein that has both NAD synthase and chaperone function, was shown to interact with phosphorylated hTau oligomers *in vivo* and promote the ubiquitination and clearance of toxic hTau species [53]. In contrast, other chaperones, *DnaJ-1*, *Csp*, and *Hsc70Cb*, have been identified unexpectedly as enhancers of hTau^V337M^ toxicity [43]. Despite the cytosolic subcellular localization of Tau, expression of hTau^WT^, hTau^R406W^, and hTau^E14^ in *Drosophila* brain triggers the unfolded protein response (UPR), a cell response that handles excess misfolded proteins in the secretory pathway causing endoplasmic reticulum (ER) stress [69]. Mild ER stress is protective in retinal degeneration [70]. The UPR similarly protected against tau neurotoxicity [69].

Alternative degradation processes to the ubiquitin-proteasome system include proteases and the autophagy-lysosomal pathway. Calpain A and B have been shown to cleave Tau and generate a toxic 17 kDa Tau fragment [30]. Mutations that disrupt endogenous calpain A or calpain B activity or expression of a calpain-resistant form of Tau hTau^K44Q/R230Q^ in transgenic flies abrogated Tau toxicity *in vivo* [30]. The puromycin-sensitive aminopeptidase (PSA) has also been described as a modifier of Tau toxicity but as a suppressor [22]. Although the original study described that PSA digests directly Tau *in vitro* [22, 71], this idea has been challenged more recently [72]. As shown in a *Drosophila* polyQ disease model, the protective effects of PSA may instead be mediated through activation of autophagy [73]. Several components related to the autophagy-lysosomal pathway, *Atg6*, *Vha14*, *Vha44*, *white*, *brown*, *rosy*, *dynein light chain 2*, *benchwarmer/spinster,* and *cathepsinD,* have been identified as modifiers of Tau toxicity in *Drosophila* [31, 42, 43, 46, 62]. Loss-of-function mutations of *benchwarmer/spinster*, *white, brown, *and* cathepsinD* are associated with enlarged lysosomes and enhanced hTau toxicity [31, 46, 62]. Loss of *cathepsinD* is also associated with caspase activation and generation of a C-terminally truncated form of hTau, which is more toxic and less soluble [31]. This suggests that caspase cleavage of Tau may be a molecular mechanism through which lysosomal dysfunction and neurodegeneration are causally linked in Tauopathies.

### 3.4. Cell Death Pathways Associated with Tau Toxicity

Many studies have detected markers of apoptosis, such as TUNEL-positive staining, activated cleaved caspase 3, cleaved PARP, and abnormal accumulation of lamin in hTau-expressing tissue [7, 25, 29, 50, 53, 54, 66, 69]. Components of the apoptotic pathway, such as dIAP1 and FEM-1, were identified as modifiers of Tau^V337M^ toxicity [41]. In addition, overexpression of dIAP1, dIAP2, and the baculovirus caspase inhibitor p35 partially rescued the hTau-mediated rough eye phenotype [7]. These results strongly support a role for apoptosis in Tau-induced neurodegeneration in *Drosophila*. The pathways that activate apoptosis upon Tau toxicity have not been described yet. The study of *cathepsinD* mutant showed that lysosomal dysfunction could trigger caspase activation in fly Tau models [31]. Reactivation of the cell cycle downstream of Tau phosphorylation has also been shown to precede apoptosis in postmitotic neurons [29]. Fly brains overexpressing hTau^R406W^ stained positive for the cell cycle markers PCNA and PH3. Inhibition and activation of the cell cycle, respectively, reduced and increased Tau toxicity but did not affect Tau phosphorylation. This indicated that cell cycle activation is a mediator of Tau toxicity acting downstream of Tau phosphorylation. This was confirmed by the use of the phosphomimetic hTau^E14^, which induced increased cell cycle marker staining and toxicity in comparison with hTau^WT^. The link between Tau phosphorylation and cell cycle activation was shown to be mediated by the TOR (Target of Rapamycin kinase) pathway [29]. Furthermore, oxidative stress has been shown to enhance Tau-induced cell cycle activation and toxicity [54]. Genetic or pharmacological inhibition and induction of oxidative stress reduced and increased hTau^R406W^ toxicity. Oxidative stress did not act by altering Tau phosphorylation but enhanced cell-cycle activation [54].

### 3.5. Cell Signaling Pathways Modulating Tau Toxicity

Several cell signaling pathways have been shown to modulate Tau toxicity but the exact contribution of each pathway and their interactions remain to be clarified and confirmed.

#### 3.5.1. The Wingless(Wg)/Wnt Pathway

Sgg/GSK3*β*, one of the major Tau kinases, belongs to the Wingless(Wg)/Wnt pathway. GSK3*β* increased hTau phosphorylation, induced hTau aggregation, and increased hTau toxicity [7]. In the Wg/Wnt pathway, Sgg/GSK3*β* inhibits armadillo/*β*-catenin, the downstream effector with TCF. Whereas one could expect that inhibition of armadillo and dTCF also contributed to GSK3*β*-enhanced hTau toxicity, loss of *armadillo* and *dTCF* rescued hTau rough eye phenotype whereas misexpression enhanced it. This showed that (1) GSK3*β* does not exert its primary effect via the canonical Wg pathway but, rather, via direct hTau hyperphosphorylation. (2) The Wg pathway contributes to hTau toxicity, possibly through a function in apoptotic cell death in degeneration associated with hTau [7]. However, mimicking the *Wg* signaling pathway by coexpression of dishevelled, an upstream component of the pathway, with hTau^WT^ reduces Tau phosphorylation and Tau toxicity [48]. Therefore, the Wg pathway seems to be involved in Tau pathology but the role of each component of the pathway remains to be clarified.

#### 3.5.2. The Mitogen-Activated Protein Kinase (MAPK) Pathways

Several members of the MAPK family have been involved in modulating hTau toxicity. From the cell stress response pathway, the stress kinase Hemipterous(Hep)/JNK was identified as an enhancer of the hTau^V337M^-induced rough eye phenotype [41]. Overexpression of Hep/JNK also exacerbated 0N4R hTau^WT^ toxicity without affecting the phospho-deficient hTau^AP^, which suggested that Hep/JNK-induced increased hTau toxicity is mediated through hTau phosphorylation [27]. This mechanism has been involved in the stress-inducing nonlethal reduction of axonal transport [52]. Hep/JNK activation has also been detected upon oxidative stress and the extent of Hep/JNK activation correlated with the degree of hTau-induced neurodegeneration [54]. In this model, oxidative stress acted not to promote hTau phosphorylation but to enhance hTau-induced cell cycle activation [54]. Recently, a genetic screen for modifiers of 2N4R hTau toxicity identified components of the Extracellular Regulated Kinase (ERK)/MAPK and p38/MAPK pathways [42]. Loss-of-function alleles of *ksr* and *Neuroglian* (*Nrg*), both of which promote ERK signaling, suppressed hTau toxicity, suggesting that reducing ERK activity is beneficial. Overexpression of *Mekk1*, a MAP3K that leads to the phosphorylation and activation of p38/MAPK, was found to strongly enhance hTau toxicity. Cross-talk between these signaling pathways was shown and may also include interactions with the GSK3*β* signaling pathway [42]. Interestingly, in this study, some results suggested that hTau could have a signaling function and be able to regulate its own kinases. In addition, overexpression of 2N4R hTau^S11A^ was previously shown to increase the activated form of GSK-3*β* (Sgg Y^214^) in comparison with 2N4R hTau^WT^ [23]. As suggested by Ambegaokar and coworkers, if Tau has the ability to regulate its own kinases, and if this regulation is phosphorylation-dependent, this would broaden our understanding of the role of Tau phosphorylation, which to date has been primarily associated with reduced microtubule-binding affinity [42].

#### 3.5.3. The TOR Pathway

It has been shown that the TOR pathway links phosphorylated hTau to a toxic cell cycle activation in postmitotic neurons [29]. Overexpression of hTau^R406W^ upregulated S6K phosphorylation, the target of TOR kinase. Pharmacological or genetic inhibition of *TOR* reduced hTau^R406W^- and hTau^V337M^-associated toxicity. The TOR-induced increase in Tau toxicity was suppressed by inhibiting cell cycle activation.

#### 3.5.4. The DNA Repair Pathway

Recently, kinases from the DNA repair pathway have been involved in a toxic phosphorylation of Tau [28]. It has been shown that the *Drosophila* DNA damage-activated Checkpoint kinase2 (Chk2) phosphorylates hTau at Ser262 and enhances hTau toxicity [28]. In addition, the DNA repair response was increased by A*β*42 [74]. It is thus possible that the DNA repair pathway induced by A*β* triggers Tau phosphorylation and toxicity in the pathogenesis of AD.

#### 3.5.5. The Janus Kinase (JAK)/Signal Transducer and Activator of Transcription (STAT) Pathway in Glia

In glia, the JAK/STAT signaling pathway has been shown to be protective against Tauopathy [50]. Expression of 0N4R hTau in glial cells using the *repo* driver resulted in aged-dependent hTau phosphorylation, hTau aggregation, formation of hTau tangles and glial, and neuronal cell death. These phenotypes were associated with a progressive loss of JAK/STAT signaling. In addition, inhibiting or activating the JAK/STAT signaling pathway in glia enhanced and suppressed cell death in fly brain expressing hTau in glia [50]. The protective effect of the JAK/STAT pathway would occur downstream of Tau phosphorylation as modulating the JAK/STAT pathway did not change hTau phosphorylation [50].

### 3.6. Tau and APP/A*β*

Human A*β*42 and *Appl*, the *Drosophila* ortholog of APP, have been shown to synergistically increase Tau toxicity in *Drosophila*. Coexpression of bTau and *Appl* under the *ApplG1a* driver synergistically induced axonal transport defects in larval motor neurons, lethality at the pharate adult stage, and a juvenile phenotype at the adult stage [51]. A*β*42 expression strongly increased the rough eye phenotype of hTau-expressing flies, as well as vacuolization and the number of TUNEL-positive cells in the brain [66]. A*β*42 coexpression also exacerbated Tau-mediated disruption of axonal transport and synaptic structures, leading to locomotor defects and reduced lifespan [48]. This interaction shows that *Drosophila* is a relevant model for AD. Furthermore, studies in *Drosophila* gained insight into the synergistic link between A*β*42 and Tau. Double-transgenic flies coexpressing A*β*42 and either hTau^AP^ or hTau^E14^, the phospho-deficient and phosphomimetic hTau constructs, showed no clear changes in retinal toxicity, suggesting that the interaction between A*β*42 and hTau requires intact Ser/Thr phosphorylation sites on Tau [66]. Coexpression of hTau and A*β*42 increased hTau phosphorylation and treatment of flies coexpressing hTau and A*β*42 with LiCl suppressing the exacerbating effect of A*β*42 [48]. This suggests that GSK3*β* may be involved in the mechanism by which A*β*42 and hTau interact, potentially through hTau phosphorylation, to cause neuronal dysfunction [48]. Another mechanism has been proposed and involved the Checkpoint kinase2 (Chk2) from the DNA repair pathway [28, 74]. Coexpression of A*β*42 and hTau resulted in increased hTau phosphorylation at several sites including Ser262 and increased hTau toxicity. Mutating Ser262 to Ala prevented hTau phosphorylation at this site and alleviated A*β*42-induced Tau toxicity [74]. Ser262 is a target site of the DNA damage-activated kinase Chk2 [28]. A number of genes involved in the DNA repair pathway like Chk2 are increased in A*β*42 fly brains, and the induction of a DNA repair response is protective against A*β*42 toxicity [74]. These results suggest that activation of DNA repair pathways is protective against A*β*42 toxicity but may trigger hTau phosphorylation and toxicity in AD pathology [74].

### 3.7. Cell- and Isoform-Specificity of Tau Toxicity and Dysfunction

Discrepancies between studies suggest that mechanisms of Tau toxicity may be different depending on cell-types and Tau isoforms. A striking example comes from the phosphomimetic and phospho-deficient Tau forms (see Section 3.1). The phosphomimetic hTau^E14^ is strongly toxic in the developing eye and in the mushroom body neuroblasts, in which the phospho-deficient hTau^AP^ is not [24, 27, 29, 66], The opposite is true for vesicular motion in motor neuron axons, neurohormone release, and animal survival [49]. This is probably due to the state of differentiation of the Tau-expressing neurons and the critical role of axonal transport in these neurons. It was also shown that the phosphorylation status and stability of Tau depend on the subneuronal population in which Tau is expressed [75].

Besides cell-type specificity, hTau isoforms, FTD-related hTau mutant forms, and Tau orthologs seem to have toxic specificities despite common properties. The Skoulakis lab has compared the toxicity of different Tau isoforms in the embryonic neuroblasts that generate the mushroom body neurons, and the functional consequence on learning ability in the adult flies [24]. They observed that expression of 0N4R and 2N4R hTau neuroblasts strongly affected the development of mushroom body neuroblasts leading to the loss of mushroom bodies in the adult. Expression of the FTD-related 0N4R hTau^R406W^ and hTau^V337M^ were mildly toxic, whereas dTau, bTau, and 0N3R hTau were not toxic. By comparing bTau and hTau sequences, they identified two phosphorylation sites, Ser238 and Thr245, which were specific to hTau. Mutation to alanine of these sites fully suppressed hTau toxicity in mushroom bodies neuroblasts [24]. Hence they were able to determine the difference between bTau and hTau that is responsible for their specific effects in mushroom bodies neuroblasts. Other examples of isoform specificity have been reported outside the mushroom bodies. In motor neurons, hTau^V337M^ did not alter axonal transport in comparison with hTau^WT^ but rather affected NMJ morphology [43]. Overexpression of either dTau or hTau in the retina resulted in a similar rough eye phenotype. However, coexpression of PAR-1 with dTau led to lethality, whereas coexpression of PAR-1 with 0N4R hTau had little effect on the rough eye phenotype [39] and Par-1 coexpression increased 2N4R hTau toxicity in the eye [23]. The origin of the differences between these isoforms still need to be explored. It has to be noted that possible genetic interactions between ectopic hTau and endogenous dTau should also be considered. Endogenous dTau expression has been shown to modulate bristle loss in the fly notum induced by hTau^WT^ [47]. The level of endogenous dTau expression may participate in the cell-type specificity.

The work on the different isoforms led the Skoulakis lab to highlight the distinction between Tau toxicity and Tau dysfunction (for reviews on this subject [76, 77]). Whereas expression of 0N4R hTau and 2N4R hTau was toxic and induced loss of mushroom bodies, expression of bTau and dTau did not affect the structure of the mushroom bodies but affected cognitive function [24]. Other phenotypes reported by other group are also more related to toxicity or dysfunction. Eye roughness, lethality, and decreased lifespan represent toxicity, whereas some axonal transport, behavioural, or synaptic defects represent dysfunction and were reported without toxicity [55]. The next question will be to understand what regulates Tau toxicity and Tau dysfunction. *Drosophila* has already given some cues. Phosphorylation of Tau may be a good candidate. Phosphorylation of Ser238 and Thr245 has been shown to be responsible for hTau toxicity in mushroom bodies neuroblasts [24]. Phosphorylation of Tau by PAR-1 has been shown to be toxic in the eye [25], whereas phosphorylation of Tau by GSK-3*β* seemed to reduce microtubule binding rather than be toxic and thus be related to dysfunction [23].

## 4. Genetic Screens in *Drosophila*: Perspectives

### 4.1. Forward Genetic Screens

To date, three forward genetic screens for hTau modifiers have been published, all using the hTau-associated rough eye as a readout for toxicity [41–43]. The first two screens reported were highly similar in design. Both screens were performed using the FTD-associated hTau^V337M^ mutant form of Tau and both screens screened transposon insertions inducing misexpression [41, 43]. The transposon insertions, P{EP} and P{Mae-UAS-.6.11}, can induce both gain- and loss-of-function phenotypes, depending on the orientation of the insertion and the presence of a GAL4 driver. Although the screens were highly similar in design, the outcomes were considerably different likely because of differences in the screened transposon collections. The screen by Shulman and Feany identified kinases and phosphatases as most represented interactors [41]. Among these, genes previously shown to affect hTau phosphorylation were found to modify the rough eye phenotype. They identified *par-1*, the *Drosophila* homolog of MARK as a suppressor of hTau toxicity. Furthermore, two regulatory subunits of the known tau phosphatases, PP1 and PP2A, were identified. In the category of the kinases and phosphatases, *string *and *twine* were also found. Both are phosphatases, and when overexpressed, suppress Tau toxicity. This suggests that *string *and *twine* might dephosphorylate tau and hence reduce toxicity. Additionally, *thread *and CG9025 were identified. Both are inhibitors of apoptosis, and the effect of the transposon insertions is as expected. If apoptosis inhibition is enhanced, the hTau rough eye phenotype is suppressed. Other interactors hinted a role for the cytoskeleton; overexpression of *orbit*, *dfxr1,* and *cheerio* aggravated the tau-induced rough eye. The last category overlaps with the findings of Blard and colleagues in their very similar screen setup [43]. They identified *cheerio, Chd64, jaguar, *and *paxillin* as enhancers. Remarkably, all four of these genes are linked to the actin cytoskeleton. *Cheerio*, also identified in the screen of Shulman and Feany, encodes the *Drosophila *homolog of filamin. Filamins play an important role in stabilizing and cross-linking filamentous actin. A loss-of-function insertion in *Chd64*, the homolog of mammalian *transgelin-3, *was found to enhance the tau phenotype. In mammals, *transgelin-3* was shown to colocalize with filamentous actin and *α*-tubulin, but also with Tau itself and MAP2. *Jaguar* was found to be homologous to mammalian class VI Myosins, which are motor proteins who transport their cargo towards the actin minus ends [43]. Paxillin is a regulator of the Rho family GTPases, Rac and Rho, which regulate actin cytoskeletal dynamics. Other interactors identified in the screen included following transcriptional regulators, CG33097, *dumpy, *and *nab, *but also several transporters (1.28, *vha44*, *ATP*α**) were identified.

The most exhaustive screen was recently published by Ambegaokar and Jackson [42]. They screened two different P-element insertion collections. In addition to the EY collection, which can induce both gain- and loss-of-function effects, this group for the first time screened a loss-of-function collection consisting of 920 genomically mapped lethal P-elements. In total, 1905 lines were screened and 37 modifiers of tau toxicity were identified. To exclude suppressors that act on general apoptosis, each of the suppressors was tested for inhibition of apoptosis by crossing them to flies eye specifically overexpressing an inducer of apoptosis, *hid*. The modifiers were then tested for effects on the Tau phosphorylation state. No consistent effects were observed among enhancers or suppressors. To quantify the effects on eye morphology, volume calculations were performed on eyes of both enhancers and suppressors. The authors could show that for all suppressors eye volume was increased, the opposite was shown for the enhancers. Using the software tool “Endeavour-highfly,” the investigators constructed a network that extrapolates the findings and suggests new pathways, cellular processes, and genes that might also be involved in pathology. A network with the predicted associations with statistical predictions *P* < 0.001 shows an interesting role for RNA processing, lysosomal degradation pathways, and, as expected, kinases and phosphatases. Five of the *in silico *predicted genes were tested for their ability to modify tau pathology *in vivo*. Both Tom34, a mitochondrial protein, and Csul, a protein involved in RNA trafficking, enhanced Tau pathology when gene function is reduced. Overexpression of both genes could induce a substantial rescue. A gene involved in RNA catabolism, *armi, *enhanced Tau pathology in both loss-of-function and gain-of-function approaches. *Upf1*and *Tom20*, involved in, respectively, RNA catabolism and mitochondrial function, did not exert an effect on external eye morphology of flies overexpressing hTau. This shows that the applied software prediction tool is able to successfully identify genetic interactors of hTau, based on results of a forward genetic screen.

### 4.2. Integration of Mammalian Genome-Wide High-Throughput Experiments with Functional Validation in Drosophila

Genome-wide high-throughput experiments such as genetic genome-wide association studies (GWAS) and transcriptomics have emerged during the last years as powerful strategies to identify novel biological disease pathways. However, given the vast amount of novel data generated in these experiments and the fact that the function of many of the identified genes or loci are not known, the biological interpretation of these studies is difficult. Recently, two very interesting strategies tackling this problem were published. The first study combines transcriptome analysis of mice overexpressing Tau with functional validation in *Drosophila* [22]. The second study starts with target identification in an AD GWAS study, followed by validation in the fly [78]. In both studies, *Drosophila* plays a central role in the functional validation of targets found in mice or patients.

Karsten and colleagues analyzed transcriptomes of different brain regions of mice overexpressing the most common FTD-associated mutant hTau^P301L^. These mice phenocopy major pathological hallmarks of tauopathy including neurodegeneration in the spinal cord and cortex. Neurodegeneration becomes apparent when the mice reach 7 to 9 months of age but transcriptome analysis was performed at 6 months of age when no major pathological phenotypes are present, but low amounts of phosphorylated Tau can already be detected. In total, 31 probe sets were found to be differentially regulated in Tau mice compared to wild-type controls. An interesting hit was the puromycin-sensitive aminopeptidase (PSA) for which no neuroprotective role had previously been suggested. To further investigate the role of PSA and other genes, mutant alleles of the *Drosophila *homologs were tested in a fly tau model. Loss of-function of PSA enhanced while overexpression suppressed Tau toxicity. To further validate PSA as a causal gene in neuroprotection, PSA abundance was evaluated in both FTD patients and controls. Interestingly, in both patients and controls, PSA expression was fivefold higher in the cerebellum than in the cortex and a significant increase in PSA was found in FTD patients compared to controls [37].

Recently, the first study combining an AD GWAS study with functional validation in *Drosophila* was published [33]. The study started with a GWAS on an autopsy cohort of 227 individuals who were nondemented at recruitment. All individuals were yearly evaluated clinically. The GWAS identified 22 gene loci suggestive of being associated with AD (*P* < 10^−3^). Of these 22 candidate genes identified, 19 genes had a clear homolog in *Drosophila*. In the second step, these genes were tested in a fly model that uses the Tau-induced rough eye as a read-out. Using a transgenic RNAi approach, each of these 19 genes were knocked down in flies overexpressing hTau^V337M^. If fly strains were available, overexpression of the identified candidate genes was also tested. Six genes, when knocked down, displayed a clear genetic interaction with Tau, *B-spec*, *fne*, *Glut1*, *hs6st*, *dlg,* and *slit*. For three of them, the opposite interaction was observed with overexpression lines. When combined with statistically more powerful GWAS datasets, functional validation of GWAS hits using Tau and A*β* toxicity readouts in *Drosophila* will likely be more powerful and informative.

## 5. Conclusion

In this paper, we have attempted to give an overview of the many technical possibilities the *Drosophila* system offers to study the molecular, cellular, and physiological mechanisms by which Tau causes neuronal degeneration and dysfunction. The available studies reveal that Tau is involved in a wide range of cellular and biochemical processes. Regarding the role of phosphorylation of Tau in neuronal degeneration, studies in *Drosophila* suggest that this link is highly complex and possibly less important than generally accepted. The most innovating results from *Drosophila* have and will continue to come from (1) unbiased high-throughput forward genetic screens that identify modifiers of Tau neurotoxicity and (2) studies that address the normal function the dTau gene. Finally, linking unbiased *Drosophila* to human genetics will likely identify important molecular mechanisms involved in Tau-mediated neuronal degeneration.

## Figures and Tables

**Table 1:** Tau constructs available in *Drosophila. *

Constructs	Type	Origin
*Drosophila* *constructs *		
UAS-dTau	Wild-type	Mershin et al. 2004 [32]
UASp-dTau^A^:mGFP6	Fusion	Doerflinger et al. 2003 [33]
UAS-dTau:1D4	Tagged form	Feuillette et al. 2010 [34]

*Bovine constructs*		
UAS-bTau	Wild-type	Ito et al. 1997 [18]
bTau^1–382^:GFP	Fusion	Micklem et al. 1997 [35]
UAS-bTau:GFP	Fusion	Murray et al. 1998 [19]
UAS-bTau:lacZ	Fusion	Callahan and Thomas 1994 [20]

*Human 0N3R construct*		
UAS-hTau		Williams et al. 2000 [21]

*Human 2N4R constructs*		
gl-hTau	Wild-type	Jackson et al. 2002 [7]
gl-hTau^P301L^	FTDP17 mutation	Karsten et al. 2006 [22]
UAS-hTau		Chatterjee et al. 2009 [23]
gl-hTau^S2A^ (S262A/S356A)	Phospho-deficient	Chatterjee et al. 2009 [23]
UAS-hTau^S2A^ (S262A/S356A)	Phospho-deficient	Chatterjee et al. 2009 [23]
gl-hTau^S11A^		
(S46A/T50A/S199A/S202A/		
S205A/T212A/ S214A/T231A/	Phospho-deficient	Chatterjee et al. 2009 [23]
S235A/S396A/S404A)		
UAS-hTau^S11A^		
(S46A/T50A/S199A/S202A/		
S205A/T212A/S214A/T231A/	Phospho-deficient	Chatterjee et al. 2009 [23]
S235A/S396A/S404A)		
UAS-hTau:FLAG	Tagged form	Kosmidis et al. 2010 [24]
UAS-hTau^STA^:FLAG (S238A/T245A)	Tagged form Phospho-deficient	Kosmidis et al. 2010 [24]

*Human 0N4R constructs*		
UAS-hTau	Wild-type	Wittmann et al. 2001 [6]
UAS-hTau^V337M^	FTDP17 mutation	Wittmann et al. 2001 [6]
UAS-hTau^R406W^	FTDP17 mutation	Wittmann et al. 2001 [6]
UAS-hTau^R406W S2A^ (S262A/S356A)	FTDP17 mutation Phospho-deficient	Nishimura et al. 2004 [25]
UAS-hTau^R406W S202A^	FTDP17 mutation Phospho-deficient	Nishimura et al. 2004 [25]
UAS-hTau^T111A/T153A^	Phospho-deficient	Steinhilb et al. 2007 MBC [26]
UAS-hTau^T175A/T181A^	Phospho-deficient	Steinhilb et al. 2007 MBC [26]
UAS-hTau^T199A/T217A^	Phospho-deficient	Steinhilb et al. 2007 MBC [26]
UAS-hTau^S202A/S205A^	Phospho-deficient	Steinhilb et al. 2007 MBC [26]
UAS-hTau^T212A^	Phospho-deficient	Steinhilb et al. 2007 MBC [26]
UAS-hTau^S214A^	Phospho-deficient	Steinhilb et al. 2007 MBC [26]
UAS-hTau^T231A/S235A^	Phospho-deficient	Steinhilb et al. 2007 MBC [26]
UAS-hTau^S262A^	Phospho-deficient	Iijima-Ando et al. 2010 [28]
UAS-hTau^S396A/S404A^	Phospho-deficient	Steinhilb et al. 2007 MBC [26]
UAS-hTau^S422A^	Phospho-deficient	Steinhilb et al. 2007 MBC [26]
UAS-hTau^AP5^		
(S202A/S205A/T212A/	Phospho-deficient	Steinhilb et al. 2007 MBC [26]
T231A/S235A)		
UAS-hTau^AP^		
(T111A/T153A/T175A/T181A/		
S199A/S202A/S205A/T212A/	Phospho-deficient	Steinhilb et al. 2007 JNR [26]
S214A/T217A/T231A/S235A/		
S396A/S404A/S422A)		
UAS-hTau^E14^		
(T111E/T153E/T175E/T181E/		
S199E/S202E/S205E/ T212E/	Phosphomimetic	Khurana et al. 2006 [29]
T217E/T231E/S235E/S396E/		
S404E/S422E)		
UAS-hTau^K44Q/R230Q^	Calpain-resistant	Reinecke et al. 2011 [30]
UAS-hTau^44–230^	Calpain 17kDA proteolytic fragment	Reinecke et al. 2011 [30]
UAST-hTau^1–421^	C-terminally truncated Tau	Khurana et al. 2010 [31]

**Table 2:** Readouts of Tau toxicity in *Drosophila*.

Organ/Tissue/Cells	Phenotypes	Promoters/Drivers
Eye	Roughness	GMR
	Photoreceptor cell viability	Gl
		Sev

Notum	Loss of bristles	Eq

Nervous system	Lethality	Elav
	Shortened lifespan	Appl
	Brain vacuolisation	Repo
	TUNEL positive cells	Gl
	Activated caspase3 positive cells	C472
	Loss of olfactory Learning and memory	C772

Motor neurons	Axonal transport defects	D42
	Locomotor deficits	OK6
	“juvenile” phenotype: loss of wing	Elav
	expansion and cuticle tanning	Burs12
		Appl
